# Older adults with lower autobiographical memory abilities report less age-related decline in everyday cognitive function

**DOI:** 10.1186/s12877-020-01720-7

**Published:** 2020-08-26

**Authors:** Carina L. Fan, Kristoffer Romero, Brian Levine

**Affiliations:** 1 Department of Psychology, University of Toronto, 4th floor, Sidney Smith Hall, 100 St. George Street, Toronto, ON M5S 3G3 Canada; 2 Rotman Research Institute, Baycrest, 9th floor, Kimel Family Building, 3560 Bathurst Street, Toronto, ON M6A 2E1 Canada; 3 Department of Medicine (Neurology), University of Toronto, 27 King’s College Circle Toronto, Toronto, ON M5S 1A1 Canada

**Keywords:** Autobiographical memory, Aging, Individual differences

## Abstract

**Background:**

Individuals differ in how they remember the past: some richly re-experience specific details of past episodes, whereas others recall only the gist of past events. Little research has examined how such trait mnemonics, or lifelong individual differences in memory capacities, relate to cognitive aging. We specifically examined trait episodic autobiographical memory (AM, the tendency to richly re-experience episodic details of past events) in relation to complaints of everyday cognitive functioning, which are known to increase with age. Although one might predict that individuals reporting higher trait-level episodic AM would be resistant to age-related decline in everyday function, we made the opposite prediction. That is, we predicted that those with lower trait-level episodic AM would be better equipped with compensatory strategies, practiced throughout the lifespan, to cope with age-related memory decline. Those with higher trait-level episodic AM would have enhanced sensitivity to age-related cognitive changes due to their tendency to rely on their perceived above-average memory function.

**Methods:**

We tested these predictions in 959 older adults aged 50–93 using online subjective and objective measures of memory and cognitive function. Our key measures of interest were the Survey of Autobiographical Memory, a measure of autobiographical memory abilities; and the Cognitive Failures Questionnaire, a measure of everyday cognitive function.

**Results:**

In keeping with our prediction, we found that complaints of day-to-day memory slips and errors (normally elevated with age) remained stable or even decreased with age among those reporting lower trait-level episodic AM, whereas those reporting higher trait-level episodic AM reported the expected age-related increase in such errors. This finding was specific to episodic AM and not observed for other autobiographical memory capacities (e.g., semantic, spatial). It was further unaccounted for by response bias or objectively assessed cognitive abilities.

**Conclusions:**

Congenitally low trait-level episodic AM may paradoxically confer a functional advantage in aging. This could be due to well-developed non-episodic strategies not present in those with higher abilities, who are more sensitive to age-related memory decline attributable to medial temporal lobe changes. Our findings emphasize the importance of considering individual differences when studying cognitive aging trajectories.

## Background

Normal aging is accompanied by cognitive changes across many domains, especially episodic memory, or memory for details with specific spatiotemporal contexts [1–3]. Given how essential healthy memory function is to the completion of everyday tasks, episodic memory impairments can translate directly into a decline in daily function for older adults [4]. However, research in this area has been hampered by two primary limitations: reliance on laboratory-based tests of memory that do not fully capture real-life memory abilities, and failure to consider individual differences.

While many studies have focused on using stimuli such as word lists to measure specific aspects of human memory, performance on such tasks does not necessarily generalize to memory use in the real world [5, 6]; as such, researchers have increasingly turned their attention to the study of autobiographical memory (AM). AM can be conceptualized as a system of knowledge related to the self that interacts with active goals and self-image in the act of remembering [7]. As such, AM encompasses both specific details from personally experienced past events, as well as personal factual knowledge [8], corresponding to Tulving’s [9] conceptualization of episodic and semantic memory respectively. Measures of episodic memory typically show greater decreases in aging than do measures of semantic memory [10–12].

However, much of the existing research examining age-related episodic memory change aggregates across individuals to assess memory performance, ignoring crucial individual differences^1^ in cognitive aging trajectories [2, 13]. Trait AM, or the style in which individuals tend to process AM, can be considered a congenital ability that remains relatively stable throughout the lifetime [14], analogous to traits such as personality or intelligence. We conceptualize trait episodic AM as reflecting an individual’s long-standing ability to recollect episodic details of past events with an accompanying strong sense of autonoetic subjective recollection [15]. Trait episodic AM may decouple from episodic memory performance as measured by a laboratory task, which is a snapshot of an individual’s putative episodic memory function at a moment in time. Individuals with low trait episodic AM may be able to produce episodic-like details without experiencing the autonoetic awareness that is a hallmark of episodic remembering, or true episodic details about a few memorable events, but their ability to do so is limited and they focus on gist-level analysis when spontaneously reflecting on past events. Such individuals can perform normally on laboratory-based episodic memory tasks by using non-episodic strategies such as relying on rehearsed cues or familiarity in the absence of recollection [16]. Like other traits, self-reported AM traits are expected to be stable with age, even as episodic memory performance declines.

Researchers have recently begun to investigate these trait-level individual differences in AM capacity empirically [17], finding corresponding neural correlates in medial temporal lobe (MTL) and prefrontal function [18, 19]. These findings suggest that AM abilities may be conceptualized as a continuum on which people differ at the trait level [14]. Accordingly, individuals at both extremes of AM abilities have been identified: those with Highly Superior Autobiographical Memory (HSAM) can richly and accurately recall almost all personal past events [20], whereas those with Severely Deficient Autobiographical Memory (SDAM) are unable to vividly re-experience past events and have difficulty recalling specific episodic details, despite normal learning and memory for facts [16], a syndrome unaccounted for by clinical pathology.

To our knowledge, no work has examined how trait-level individual differences moderate age-related cognitive change. Intuitively, one might expect that strong trait episodic AM abilities pose a benefit in aging, as an extensive body of work has documented the contribution of episodic memory processes to many other functions in both younger and older adults [21–24]. As such, one might expect that individuals who tend not to recall past events with such detail may be at a disadvantage with respect to everyday functioning.

However, much of the research focusing on the role of episodic memory processes in other cognitive domains assessed lab-based episodic memory task performance, rather than trait-level individual differences in episodic AM. We hypothesized that individuals with higher trait episodic AM tend to rely more on episodic memory to carry out daily tasks and will thus be more heavily impacted should their episodic memory begin to decline with age [14]. Individuals with lower trait episodic AM and a lifetime of practice performing everyday tasks without the use of strong episodic processes may find that their daily function is relatively unimpacted by age-related changes in episodic memory performance. SDAM individuals demonstrate little evidence of functional impairment in day-to-day life [16], suggesting that they have developed non-episodic mnemonic strategies. Individuals with low trait episodic AM may be better able to recruit non-episodic processes in their daily function due to experience, innate differences in brain structure and function, or both—in other words, these individuals may have better task processing efficiency or a sort of cognitive reserve that allows them to maintain daily function despite declining episodic memory performance [19, 25, 26]. Those reliant upon congenitally strong episodic memory abilities may be relatively lacking in these strategies and therefore must newly adjust to age-related changes in memory performance.

The aim of the present study was to investigate how trait AM moderates age-related decreases in older adults’ everyday cognitive functioning. We tested our hypothesis in a sample of almost one thousand community-dwelling adults aged 50–93 tested online. We measured trait AM with a validated questionnaire, the Survey of Autobiographical Memory (SAM [17]). Everyday cognitive function was assessed with the Cognitive Failures Questionnaire (CFQ [27]). Our test battery was augmented by additional performance-based measures of memory and cognition. We expected that trait AM as assessed by the SAM would reflect stable individual differences in AM and would thus be unrelated to age. Consistent with the body of work investigating cognitive aging, we expected that age would be associated with a decline in performance on memory and cognitive function as measured by objective tests. Finally, and critically, we hypothesized that individual differences in trait episodic AM would moderate the relationship between aging and everyday cognitive function as assessed by the CFQ, such that higher AM capacity would be associated with greater evidence of age-related decreases in everyday functioning—a prediction opposite to expectation based on shared variance in self-report. Such a finding would suggest that lower trait AM paradoxically protects against the effects of age-related memory changes on everyday functioning, possibly through the lifelong practice of strategies that bypass reliance on the brain’s age-sensitive episodic memory system.

## Methods

### Participants

Participants were recruited online through the Canadian Association of Retired Persons (CARP). Members of CARP were invited via email to participate and compensated with a $10 gift card. All participants gave written informed consent at the start of the survey. The study procedure was approved by the Baycrest Research Ethics Board.

#### Exclusions

Two thousand six hundred twenty-six survey responses were recorded. Six hundred twenty-nine responses were blank in the fields of interest (i.e., only had data such as location coordinates, but not for our questionnaires) and were removed from the dataset. For multiple survey responses listed with the same email address, only the first response was kept for analysis (155 emails were associated with two or more responses). In addition, responses were removed if they were completed in an implausibly short length of time (i.e., less than 3 min, *n* = 76). Participants reporting fewer than 9 or greater than 26 years of education were excluded (*n* = 25), as well as participants under the age of 50 (*n* = 28).

We included 3 “catch” questions as part of the online survey to ensure participants were paying attention and were human (rather than bots), in which they were asked to select a specific point on a Likert scale (e.g., “For this question, please select ‘Totally agree’.”). We also included a text catch in which participants had to answer “What is 3 plus seven” in a text box. Responses that failed one or more of the catch questions were excluded from analyses (*n* = 519).

After these preliminary cleaning steps, any participants who endorsed a history of major neurological conditions were excluded from the data set (mild cognitive impairment, *n* = 61; dementia, *n* = 7; transient ischemic attack or stroke, *n* = 75; seizures, *n* = 29; multiple sclerosis, *n* = 9; moderate to severe traumatic brain injury, *n* = 31; brain tumour, *n* = 12, brain surgery, *n* = 17; Parkinson’s, *n* = 5; amnesia, *n* = 2; brain aneurysm, *n* = 3). Furthermore, participants who scored 20 or higher on the Geriatric Depression Scale (GDS [28]), indicating severe depressive symptoms [29], were excluded (*n* = 57). Finally, participants who were missing data in any of the measures and questionnaires of interest to the present study were excluded (*n* = 128).

After all exclusions, our sample comprised 959 older adults aged 50–93 years (*M* = 68.34, *SD* = 7.09). On average, the sample had 15.58 years of education (*SD* = 2.59), and it included 606 females, 351 males, and 2 individuals who indicated “Other/Prefer not to answer” with respect to gender.

At the end of our online survey, participants were redirected to Cambridge Brain Science’s (CBS) web-based platform to complete four short neurocognitive tests. Four hundred thirty-six participants completed this portion of the study; thus, all reported analyses involving CBS tasks include only this smaller sample. The sample comprised 272 females, 163 males, and 1 other gendered, with ages ranging from 50 to 90 (*M* = 67.14, *SD* = 6.74) and an average of 15.68 years of education (*SD* = 2.54).

Although we did not formally screen for mental status changes indicative of a neurodegenerative process, we note that our test battery required significant interaction with a computer interface and completion of moderately complex timed tasks that, in our experience, could not be successfully completed by someone with significant cognitive impairment. That said, we cannot rule out the presence of neurodegenerative or other pathological processes in our sample.

### Measures

All our online materials, except the face-name and CBS tasks, can be found on the Open Science Framework (OSF [30]). Commercial versions of the CBS tasks are publicly available online [31].

The SAM [17] is a validated self-report questionnaire that assesses trait mnemonics in four domains: episodic (e.g., “When I remember events, I remember a lot of details”), semantic (e.g., “I can learn and repeat facts easily, even if I don’t remember where I learned them”), spatial (e.g., “In general, my ability to navigate is better than most of my family/friends”), and future (e.g., “When I imagine an event in the future, the event generates vivid mental images that are specific in time and place”). Total scores on each of the four memory domains are derived using a weighting algorithm on the raw item ratings, developed during the original validation of the SAM. Importantly, SAM items are phrased to reflect general abilities; with respect to the SAM-episodic items, participants are instructed to respond according to their memory for events in general, not any specific event. The SAM episodic and semantic subscales relate to patterns of resting-state functional connectivity in healthy adults, suggesting that these subscales assess trait mnemonic abilities that correspond to stable patterns of brain activity [19].

Memory test performance was assessed with a face-name recognition memory task sensitive to age-related deficits [32, 33]. Participants first saw a list of 20 face-name pairs, and then they saw the same list again in a different order. Then, participants saw another 24 face-name pairs, and they judged whether each face and name had been previously paired together or not. Eight of these pairs were intact pairs that had been previously seen; eight were recombined pairs, where both the face and name had been previously seen but not paired with each other; and eight were new pairs, where neither the face nor the name had been previously seen. Participants were required to endorse, i.e., answer “yes” to, only the intact pairs, and to answer “no” to both the recombined and new pairs.

From these 24 trials, we obtained separate measures of item and associative memory as per Troyer et al. [32]; see also [34]. Item memory scores were calculated as [proportion of intact pairs correctly endorsed – proportion of new pairs incorrectly endorsed], and they reflected memory for individual faces and names without respect to their pairings. Associative memory scores were calculated as [proportion of intact pairs correctly endorsed – proportion of recombined pairs incorrectly endorsed], and they reflected memory for the pairings between specific faces and names. All participants completed the face-name task.

We also included four tasks from the Cambridge Brain Sciences (CBS) online battery [31]: Paired Associates, Grammatical Reasoning, Rotations, and Odd One Out. Four hundred thirty-six participants completed the CBS tasks. These are short, web-based tests derived from traditional pen-and-paper tests, and they tap into short-term episodic memory, verbal reasoning, mental rotation, and deductive reasoning, respectively [35]. Except for grammatical reasoning, these tests were adaptive such that item difficulty increased or decreased depending on trial-by-trial performance.

In the paired associates task, participants were shown items concealed behind boxes in different locations of the screen, and then they had to click on the box corresponding to each item. The outcome measure was the maximum number of items that could be correctly recalled. In the grammatical reasoning task, participants evaluated a series of logical statements as true or false (e.g., “Circle is not encapsulated by square”), given an image of a circle and square. They were given 90 s to answer as many items correctly as possible. The outcome measure was the number of trials completed. In the rotations task, participants were presented with two side-by-side arrays of red and green squares and indicated whether one could be rotated to look identical to the other. They were given 90 s to answer as many items correctly as possible. The outcome measure was the highest level achieved (max = 20). In the odd one out task, participants saw nine shapes in a 3 × 3 grid which differed on various attributes (e.g., colour, size, shape) and identified the one that did not fit with any of the others. Participants were given 3 min to answer as many items correctly as possible. The outcome measure was the highest level achieved (max = 20).

The CFQ [27] is a 25-item questionnaire developed to measure cognitive slips and perceived performance while completing tasks important for everyday function that a person normally should be able to complete without error, such as keeping track of ongoing conversational topics and navigating familiar environments. Higher scores indicate more slips and thus lower function. Scores on the CFQ have been used as a measure of subjective cognitive function in older adults [36] and correspond to age-related decreases in verbal memory performance [37], lending support to its use as a measure of age-related changes in cognitive functioning in daily life.

We also collected scores on the Big Five Inventory (BFI [38, 39]), a 44-item questionnaire that assesses extraversion, openness to experience, conscientiousness, neuroticism, and agreeableness. In order to analyze whether the dispositional tendency of individuals to attribute socially desirable characteristics to themselves (halo effect [40]) played a role in the relationships between age, memory abilities, and daily function, we calculated a “halo” score by taking the average of each individual’s standardized scores on openness, extraversion, conscientiousness, and agreeableness, and then subtracting the standardized score on neuroticism.

### Statistical analysis

Data were analyzed using *R* version 3.6.2 [41]. Region of significance analyses to probe interaction effects were conducted using an online utility at *quantpsy.org* [42, 43]; this analysis is reported in more detail in the results. Analyses involving repeated measures were conducted with multilevel linear models with a random intercept for participant, unstructured covariance matrices, and restricted maximum-likelihood estimation using the *lmer* function from the *lme4* package (version 1.1–21 [44]), and we obtained Satterthwaite degrees of freedom and Type III *F* tests using the *lmerTest* package (version 3.1–1 [45]). Effect sizes are reported for general linear models as standardized *β* coefficients, and for multilevel linear models as semi-partial *R*^*2*^. Bayesian analyses were conducted in JASP version 0.9.2 [46].

Given that we were only interested in normal aging for this study, we also included scores on the GDS as a covariate in all of the following analyses to account for depression. Although people with evidence of significant depression were excluded, cognitive dysfunction associated with subclinical depressive symptoms [47] remained a potential confound. Including GDS scores did not change the interpretation of main effects and interactions of interest; as such, we report the results of analyses that that did not include the GDS, for ease of interpretation. All analysis outputs (including with GDS) can be found at our OSF link.

## Results

We first examined whether the SAM measures stable trait-level individual differences in how people tend to access AM, rather than differences in memory performance that may decline with age. As predicted, age did not correlate with any of the SAM domains: episodic *(r* = − 0.03, *p* = .28, 95% *CI* [− 0.10, 0.03]), semantic (*r* = − 0.01, *p* = .84, 95% *CI* [− 0.07, 0.06]), spatial (*r* = − 0.03, *p* = .29, 95% *CI* [− 0.10, 0.03]), and future (*r* = − 0.01, *p* = .64, 95% *CI* [− 0.08, 0.05]). Given our sample size it is unlikely that this null finding is due to insufficient power, but *p*-values nonetheless do not allow us to conclude whether statistical non-significance is due to a lack of sensitivity in the data or to evidence supporting a lack of relationship between the two variables [48]. As such, we conducted Bayesian analyses for each of the SAM domains, comparing two models: the null hypothesis (*H*_*0*_) that there is no correlation between age and SAM scores, and the alternative hypothesis (*H*_*1*_) that age and SAM are related. We used a weakly-informative prior, assigning a stretched beta prior with a width of 0.5 to the uniform distribution bounded by − 1 and 1 to account for an increased likelihood of values around Pearson’s *ρ* = 0. The estimate of the correlation coefficient *ρ* for the relationship between age and SAM-episodic scores was −.02, with a 95% credible interval of − 0.07 to 0.04, indicating that we are 95% confident that the true value of *ρ* is located within these bounds. *BF*_*01*_ for this model was 15.82, indicating that the null model was almost 16 times more likely than the alternative. Results were similar for the other SAM domains: SAM-semantic *ρ* = −.01, 95% *CI* [− 0.06, 0.04], *BF*_*01*_ = 18.04; SAM-spatial *ρ* = −.01, 95% *CI* [− 0.07, 0.05], *BF*_*01*_ = 18.12; SAM-future *ρ* = −.02, 95% *CI* [− 0.07, 0.04], *BF*_*01*_ = 14.96. The SAM thus appears to measure stable trait-level individual differences in how people tend to access AM, as predicted, rather than differences in memory performance that may decline with age.

**Fig. 1:**
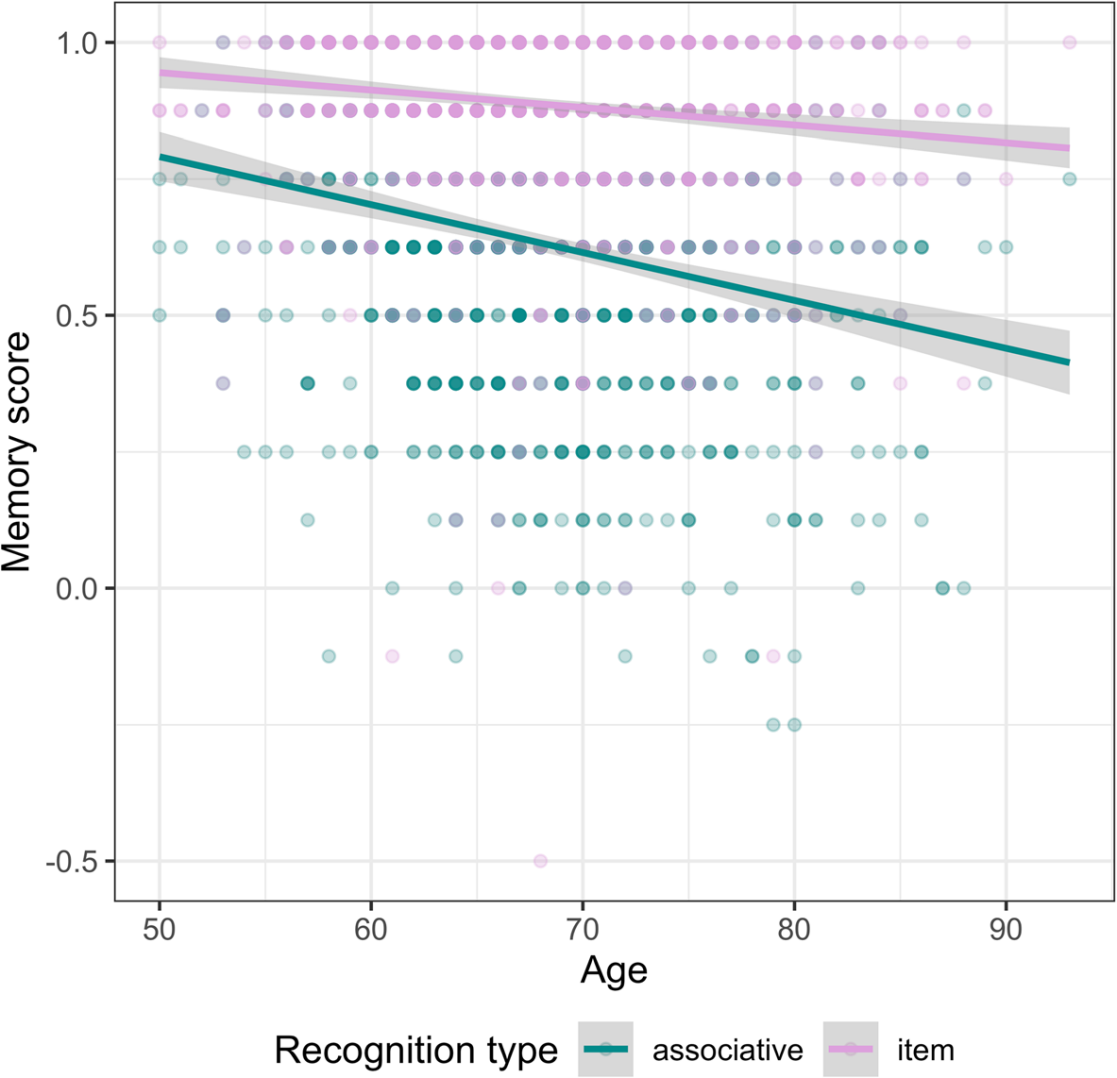
Scatterplot of age and item and associative memory scores on the face-name task

We next assessed the age-sensitivity of our mnemonic and cognitive measures as a test of the validity of our online assessment platform. Using a multilevel linear model for the face-name task, we found that age was negatively related to memory scores (*F*(1, 957) = 53.47, *p* < .001, *R*^*2*^ = .05), and associative and item scores did not differ from each other (*F*(1, 957) = 3.06, *p* = .08, *R*^*2*^ = .003). Age interacted with recognition type (associative vs. item; *F*(1, 957) = 28.50, *p* < .001, *R*^*2*^ = .03); Fig. 1 shows that associative memory scores were more sensitive to aging than were item memory scores, in line with previous research [32, 33].

Aging was also negatively correlated with all four CBS tasks: paired associates (*r* = −.28, *p* < .001, 95% *CI* [− 0.37, − 0.20]), grammatical reasoning (*r* = − 0.36, *p* < .001, 95% *CI* [− 0.44, − 0.28]), rotations (*r* = − 0.13, *p* = .005, 95% *CI* [− 0.22, − 0.04]), and odd one out (*r* = − 0.21, *p* < .001, 95% *CI* [− 0.30, − 0.12]). Overall, these results demonstrate that established findings related to aging and cognition were successfully replicated using our online platform.

Before investigating whether episodic AM as assessed by the SAM moderated the relationship between age and daily cognitive function as measured by the CFQ, we examined the direct relationship between age and the CFQ. Aging was not correlated with scores on the CFQ (*r* = .02, *p* = .63, 95% *CI* [− 0.05, 0.08]). A Bayesian correlation analysis with the same weakly-informed prior described above indicated an estimated *ρ* = .001, 95% *CI* [− 0.05, 0.06], *BF*_*01*_ = 19.15.

Our main analysis of interest involved age as the focal predictor in a linear regression with total scores on the CFQ as the outcome variable, and scores on the episodic domain of the SAM as a moderator of this relationship. The resulting model had an adjusted multiple *R*^*2*^ of .18, and standardized regression parameters are shown in Table 1. SAM-episodic scores were negatively related to CFQ scores, such that higher episodic memory abilities were generally associated with better everyday function. Critically, however, we observed an interaction between age and SAM-episodic scores, indicating that the relationship between age and function depended on episodic memory abilities. We formally probed this interaction while preserving the continuous nature of SAM-episodic scores by calculating the region of significance using the online utility at *quantpsy.org/interact* [43]. The region of significance defines the specific value of a moderator variable (in this case, SAM-episodic scores) at which the regression of an outcome variable (CFQ scores) on a focal predictor variable (age) moves from statistically non-significant to significant at *α* = .05 [42]. The upper and lower bounds of the region of significance corresponded to SAM-episodic scores of 104.62 and 78.16 respectively, with the regression of CFQ scores on age being statistically significant only outside of this range. At the upper bound SAM-episodic score, the simple slope of CFQ on age was positive (0.08), such that aging was associated with increased difficulty with cognitive function in daily life; but at the lower bound SAM-episodic score the simple slope was negative (− 0.10), indicating that older participants reported better function. As seen in the supplemental material (Table S9 and Figure S1), this finding was not altered by the inclusion of gender in the model.

**Table 1:** Regression parameters for model of age and SAM-episodic scores in predicting scores on the Cognitive Failures Questionnaire (CFQ)

	*β*	*SE*	*t*	*p*
Age	0.02	0.03	0.55	.58
SAM-episodic	−0.23	0.03	−7.44	< .001*
Age × SAM-episodic	0.09	0.03	2.73	.006*

*Note. SAM* Survey of Autobiographical Memory. *p < .05. Beta weights are standardized

**Fig. 2:**
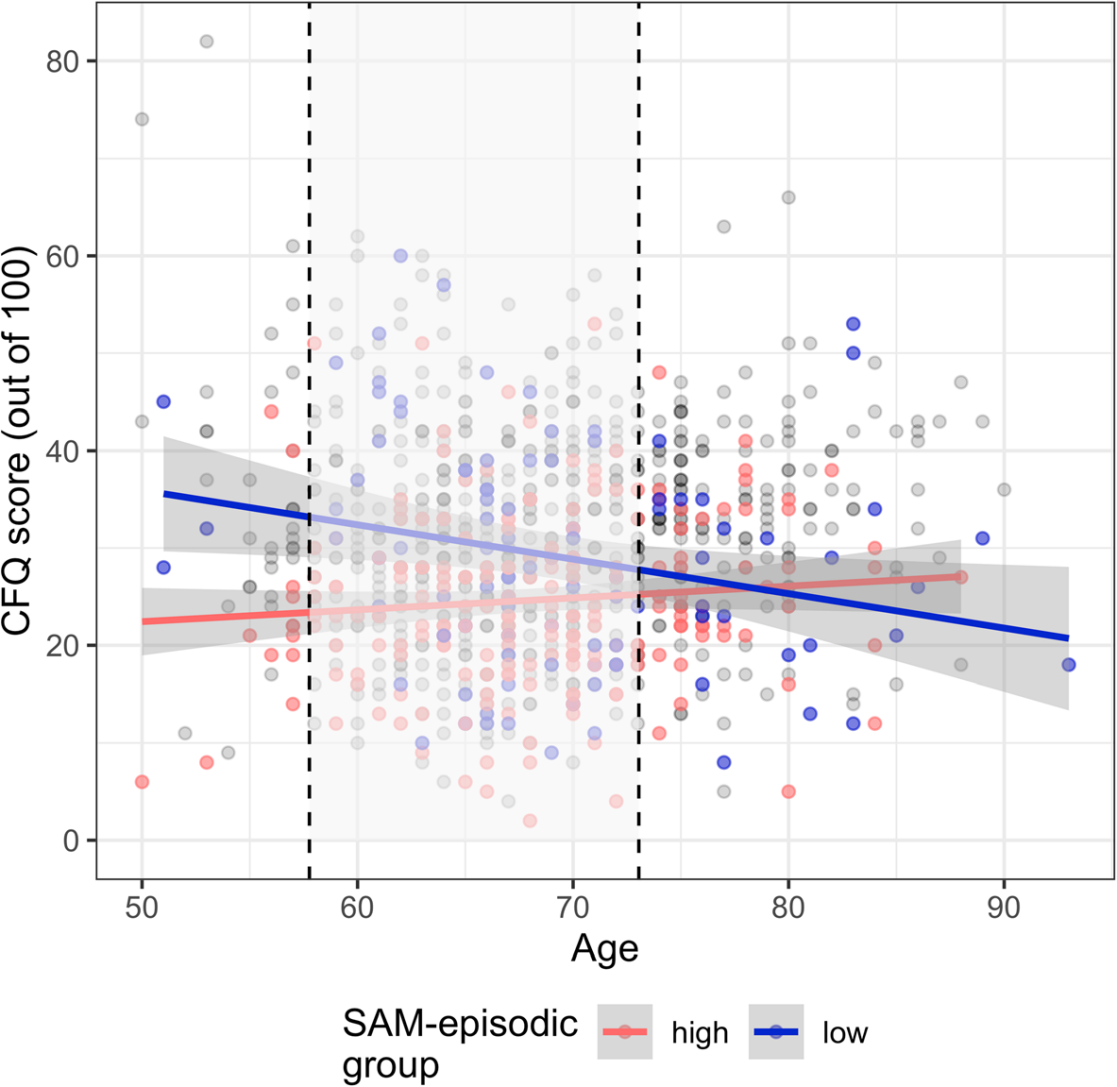
Scatterplot of age and Cognitive Failures Questionnaire (CFQ) scores, split into groups with either high or low scores on the episodic domain of the Survey of Autobiographical Memory (SAM). Groups were split based on a region of significance analysis indicating that the regression of CFQ on age was statistically significant only for participants who scored below 78.16 or above 104.62 on SAM-episodic. Individuals with scores within this range are plotted as grey points, but the corresponding (non-statistically significant) regression line is not shown. Another region of significance analysis indicated that the regression of CFQ on SAM-episodic was statistically significant only for those who were younger than 57.77 or older than 73.05 years of age, indicated by non-shaded areas outside the dashed lines

Given the symmetry of predictors in a forced-entry regression analysis, we can also interpret the interaction between age and SAM-episodic scores by treating age as a moderator in the regression of CFQ scores on SAM-episodic scores. We found a region of significance with upper and lower bounds corresponding to ages of 57.77 and 73.05, with the relationship between SAM-episodic and CFQ being statistically significant for ages outside this range. To visualize this interaction, we split participants into high (*n* = 222), low (*n* = 98), and mid (*n* = 639) SAM-episodic groups based on the region of significance bounds, and we plotted the relationship between age and subjective cognitive functioning in the low and high groups (Fig. 2).

To confirm that episodic, but not semantic, memory capacities moderated the relationship between aging and function, we ran a regression with SAM-semantic scores instead of SAM-episodic as the moderating variable in the relationship between age and the CFQ (see supplemental material, Table S1, for full regression results). There was a direct relationship between SAM-semantic scores and the CFQ such that higher semantic memory abilities were associated with better everyday function, but there was no interaction between age and SAM-semantic scores, suggesting that the relationship between age and function did not depend on individual differences in semantic memory abilities. Similar results were obtained when we tested SAM-spatial and SAM-future scores as moderators (see supplemental material, Tables S2 and S3).

Halo scores, reflecting a tendency toward positive self-appraisal, correlated positively with SAM-episodic scores (*r* = .17, *p* < .001, 95% *CI* [0.10, 0.23]) and negatively with CFQ scores (*r* = −.44, *p* < .001, 95% *CI* [− 0.49, − 0.39]), indicating that individuals who saw their personalities in a more positive light also tended to report higher episodic memory abilities and better day-to-day cognitive functioning. To assess whether this effect influenced the interaction between age and trait AM in predicting function, we ran a linear regression with halo, age, SAM-episodic, and the interaction between age and SAM-episodic as predictors of CFQ scores. The resulting model had an adjusted multiple *R*^*2*^ of .23, and standardized regression parameters are shown in Table 2.

**Table 2:** Regression parameters for model of age and SAM-episodic scores in predicting scores on the Cognitive Failures Questionnaire (CFQ), controlling for halo effects

	*β*	*SE*	*t*	*p*
Halo	−0.30	0.02	−14.57	< .001*
Age	0.07	0.03	2.42	.02*
SAM-episodic	−0.16	0.03	−5.61	< .001*
Age × SAM-episodic	0.08	0.03	2.73	.006*

*Note. SAM* Survey of Autobiographical Memory. Halo scores were calculated based on responses on the Big Five Inventory and reflect a global bias towards positive self-appraisal. *p < .05. Beta weights are standardized

Although individuals who perceived themselves in a more positive light tended to report better memory and function on both the SAM-episodic subscale and the CFQ, the interaction between age and episodic memory abilities remained over and above this effect. If our findings simply reflected a globally positive or negative response bias, then those with high SAM-episodic scores should also have higher CFQ scores. This is not what we found; rather, the interaction between SAM-episodic score and age indicates that individuals reporting higher episodic memory capacity reported *lower* everyday functioning with age, while lower trait episodic AM individuals did not.

A possible alternative explanation for the lack of age-related functional complaints among those with lower trait AM is that these individuals simply did not recall and therefore were not aware of problems in daily function. In healthy older adults, awareness of cognitive errors is intact [49]. Nonetheless, we explored whether individuals reporting low episodic AM performed worse relative to high-episodic AM individuals on our objective measures of memory and cognitive performance (i.e., face-name and CBS tasks). These analyses (see supplemental material, Tables S4, S5, S6, S7, and S8, for full regression results) revealed that SAM-episodic scores did not directly relate to cognitive performance, nor did they interact with age, indicating that low trait episodic AM as measured by the SAM was not reflective of cognitive impairment that might lead to a lack of awareness of deficits.

## Discussion

Memory is fundamental in our day-to-day lives, and age-related memory changes can impact older adults—but there is a lack of research assessing naturalistic memory at the trait level and its relationship to everyday function. We collected data online from almost one thousand community-dwelling older adults to address the role of trait mnemonics in predicting everyday function. We found that individual differences in trait episodic AM moderated the relationship between aging and declines in everyday cognitive performance: higher trait episodic AM was associated with greater levels of perceived cognitive dysfunction with advancing age. This runs counter to the intuition that having good episodic AM would be protective in aging.

The tendency to vividly re-experience past events is associated with increased connectivity between the MTLs and posterior brain regions; by contrast, the tendency to remember autobiographical events through a fact-based, semantic lens, rather than by retrieving specific episodic details, is associated with increased connectivity between the MTLs and prefrontal cortical regions [19], corresponding to a more gist-based, conceptually integrated framework (see also [18]). We reason that people with lower trait-level episodic AM have developed such non-episodic strategies over their lifetimes, and they are thus less affected by changes in brain systems supporting memory, even if they have a higher baseline rate of everyday complaints in middle age. On the other hand, those who have generally relied on high episodic AM abilities may be accustomed to relying on episodic memory to carry out everyday tasks and thus have difficulty adjusting to age-related brain changes affecting memory function, such as reductions in MTL grey matter [50–52], MTL white matter [53, 54], and changes in the function of the AM network [55, 56]. These individuals may therefore experience greater declines in everyday cognitive functioning with age. Accordingly, our region of significance analysis showed that those with higher trait episodic AM reported that everyday functional complaints increased with age. In individuals with lower trait episodic AM, endorsement of such complaints actually decreased with age; however, relatively few individuals in our sample had episodic AM abilities below the cut-off score for the region of significance, so this effect warrants further follow-up study. Overall, our findings are consistent with the cognitive reserve hypothesis that individual variability in task processing efficiency may relate to differences in susceptibility to neuropathology that disrupts cognitive performance in old age [26, 57].

Because individuals with low trait episodic AM have less ability to richly re-experience past events, they rely more on gist when spontaneously recalling memories from their lives [19]. However, they may be able to perform some episodic memory tasks well when prompted [16]—by analogy, an individual low in the trait of extraversion may tend not to engage with the outer world of people and things in general, yet they may appear to be successfully outgoing and gregarious when required to in certain social situations. However, the introverted person would be less able to sustain this style of interaction than an extraverted person would, and the introvert’s propensity to keep to themselves reflects this lower ability. Likewise, low-episodic-AM individuals’ inclination toward less episodic styles of remembering is linked to their reduced episodic ability.

Individuals with low trait episodic AM may also accomplish putatively episodic tasks in the lab using non-episodic strategies (e.g., familiarity, logistic inference), or they may produce seemingly episodic details without the accompanying sense of episodic re-experiencing. Accordingly, low SAM-episodic scores do not necessarily correspond to low episodic memory performance as measured by traditional laboratory tasks, or even some autobiographical memory tasks that can be completed via non-episodic processes [16, 58]. Indeed, a key innovation and purpose of the SAM is to tap into individual differences in naturalistic AM abilities at the trait level, which do not necessarily map onto lab-based episodic memory measures [5, 6].

We considered the possibility that individuals with lower trait episodic AM were simply unaware or amnesic for cognitive errors in daily life. Since we screened for major psychiatric or neurological conditions, awareness of cognitive errors is expected to be intact in our sample of community-dwelling older adults [49], although we acknowledge that we cannot rule out the possibility of unreported cognitive decline. However, those with low trait episodic AM reported more functional complaints than did those with high trait episodic AM (irrespective of age), and trait episodic AM as measured by the SAM was neither related to age nor to performance on our memory and cognitive tasks, reinforcing the fact that lower trait episodic memory is not a form of cognitive dysfunction. Previous research shows that those at the extremes of trait AM (HSAM and SDAM) do not show corresponding extreme scores on laboratory tasks, even when those tasks assess memory [16, 20]. Given that both trait AM and everyday cognitive function were based on self-report, we cannot rule out that subtle changes in self-appraisal with age contributed to our results; future research using objective or other-rated measures of everyday functioning can address this. On the other hand, accounting for the effect of response bias as measured by the tendency to endorse positive personality traits (i.e., the halo effect [40]) did not alter our conclusions.

To counteract issues arising from lack of supervision [59, 60] we conducted extensive cleaning and exclusion procedures to ensure that our sample comprised only older adults with reliable and valid data. Accordingly, we found that aging was negatively related to our online measures of memory and cognitive performance, replicating research demonstrating age-related cognitive decline [51, 61]. This supports the validity of our online testing platform and thus the interpretation of our other results. While it may be argued that our data cleaning criteria resulted in a sample of older adults with better-than-average daily function, our sample is much larger and no less representative than the samples of healthy older adults reported in the literature who are able to come into a laboratory for testing.

AM includes both rich episodic re-experiencing of past events, as well as semantic factual knowledge associated with the self [8]. The fact that AM abilities as measured by the SAM were not related to age supports the construct of trait AM that reflects individual differences in memory processing [14, 19]. While there is a well-established literature documenting age-related impairments in long-term memory [3, 11], we consider underlying trait mnemonics as separate from the changes in memory performance that occur with age. This hypothesis must be tested with longitudinal assessment. Nonetheless, our main finding—that higher trait AM is associated with lower everyday function with age—opposes predictions based on shared variance of self-report, and this finding is not dependent on age-invariance of trait AM.

The moderating effect of trait AM on predicting everyday function in older adults was specific to episodic memory. Semantic memory capacity as measured by the SAM was related to cognitive functioning, but the relationship between age and everyday cognitive function was constant across the range of trait semantic memory. Similar results were obtained for SAM subscales assessing spatial memory and future thinking. These results help shed further light on how different memory functions are impacted with age, and point toward the specificity of the construct of episodic AM.

The finding that lower trait AM appears to be protective in cognitive aging raises the question of whether it might also confer other seemingly paradoxical advantages. Sheldon et al. [62] demonstrated that susceptibility to visual interference effects was inversely related to visual imagery abilities: those with lower imagery were relatively unaffected by visual interference on a spatial memory task, suggesting the use of alternate strategies to achieve the same performance. Vivid episodic remembering, while promoting retention of specific details and distinctions between episodes, may inhibit the ability to extract conceptual similarities across episodes, a process necessary for generalization or making inferences [63]. As such, we speculate that lower trait episodic AM may promote generalization across episodes, one benefit of which may be protection against age-related decline. This may also speak to the nature of hypothesized compensatory strategies implemented by low trait episodic AM individuals; while our present data cannot address the specific processes underlying these strategies, it is possible that people who are less apt to use episodic memory in daily life may instead have better inferential reasoning or executive processing abilities [18].

When we examined the direct relationship between age and everyday functioning without accounting for individual differences in AM, no clear association emerged. We also found no direct relationship between aging and SAM scores, and we interpreted this to reflect the fact that the SAM measures age-invariant, trait-level AM abilities. The CFQ, on the other hand, is known to measure everyday cognitive performance that does change with age [36, 37]. When we accounted for trait-level differences in AM in examining the relationship between age and CFQ scores, we found that a predicted age-related decrease in daily function did emerge, emphasizing the importance of accounting for individual differences when examining age-related changes in memory and function, and in researching memory more broadly [2, 13]. This also addresses the concern that SAM-episodic scores were not correlated with age due to insensitivity. This measure was generally sensitive to individual differences in everyday function at younger ages in the expected direction (i.e., lower SAM-episodic scores were associated with more everyday memory problems), and of course it was sensitive to the hypothesized interaction with increasing age. Though researchers are beginning to explore individual differences in AM by studying the extreme ends of HSAM and SDAM [16, 20], there is a need for work considering how normal variations in trait mnemonics may also influence cognition in daily life.

A key limitation of the present study is its cross-sectional nature. Our hypothesis that lower trait episodic AM confers protection against age-related mnemonic changes fundamentally requires a longitudinal perspective, as it concerns a question of change over time. The present data provide support for our hypothesis nonetheless, and follow-up studies in this sample will help determine whether the pattern we observed reflects individual aging trajectories.

## Conclusion

Our findings underscore the need for behavioural and neuroimaging research to examine the mechanisms underpinning individual differences in AM abilities and associated differences in cognitive aging and declines in everyday functioning. The main outcome of interest here was function in day-to-day life, yet individual differences in AM abilities may also translate to differences in more specific areas, such as decision making or problem solving. Uncovering how variability in AM across individuals relates to age-related cognitive and functional declines will improve understanding of both AM and cognitive aging. Our findings emphasize the importance of considering individual differences when studying cognitive aging trajectories.

## Supplementary information

**Additional file 1.** Supplemental Material. Results of supplemental data analyses.

## Data Availability

The datasets generated and/or analysed during the current study are not publicly available due to restrictions from our ethics approval, but are available from the corresponding author on reasonable request. All our online testing materials, except the face-name and CBS tasks, and all our analysis scripts and outputs can be found at 10.17605/OSF.IO/7Z3WS. Commercial versions of the CBS tasks are publicly available at *www.CambridgeBrainSciences.com*.

## Abbreviations

AM: Autobiographical memory
BFI: Big Five Inventory
CFQ: Cognitive Failures Questionnaire
CBS: Cambridge Brain Sciences
GDS: Geriatric Depression Scale
HSAM: Highly Superior Autobiographical Memory
MTL: Medial temporal lobe
SAM: Survey of Autobiographical Memory
SDAM: Severely Deficient Autobiographical Memory

## Footnotes

1 We use the term “individual differences” in this study to refer to congenital differences between individuals that reflect stable traits, rather than differences between individuals across the lifespan, including aging.
