# Supplementary material for: Older adults with lower autobiographical memory abilities report less age-related decline in everyday cognitive function
Source: BMC Geriatr. 2020 Aug 26;20:308. doi: 10.1186/s12877-020-01720-7 (PMC7449056; doi:10.1186/s12877-020-01720-7)
Supplement: Supplementary file 1 — Additional file 1. Supplemental Material. Results of supplemental data analyses. [file 12877_2020_1720_MOESM1_ESM.pdf]

Older adults with lower autobiographical memory abilities report less age-related decline in everyday cognitive function

Carina L. Fan, Kristoffer Romero, and Brian Levine

Supplemental Material

To confirm that episodic, but not semantic, memory abilities moderated the relationship between aging and function, we ran a regression with SAM-semantic scores instead of SAM-episodic as the moderating variable in the relationship between age and function. The resulting model had an adjusted multiple R^2 of .11, and the results are shown in Table S1.

Table S1

Regression parameters for model of age and SAM-semantic scores in predicting scores on the Cognitive Failures Questionnaire (CFQ)

	β	*SE*	*t*	*p*
Age	0.01	0.03	0.39	.70
SAM-semantic	-0.33	0.03	-10.70	< .001*
Age × SAM-semantic	0.02	0.03	0.79	.43

Note. SAM = Survey of Autobiographical Memory. **p* < .05.

We also explored whether scores on the remaining SAM domains might moderate age-related changes in daily function. Scores on neither the spatial (Table S2) nor future (Table S3) domains of the SAM interacted with age to predict CFQ scores, pointing towards the specificity of the construct of episodic AM.

Table S2

Regression parameters for model of age and SAM-spatial scores in predicting scores on the Cognitive Failures Questionnaire (CFQ)

	β	*SE*	*t*	*p*
Age	0.008	0.03	0.26	.79
SAM-spatial	-0.26	0.03	-8.22	< .001*
Age × SAM-spatial	0.03	0.03	1.08	.28

Note. SAM = Survey of Autobiographical Memory. *$p < .05$.

Table S3

Regression parameters for model of age and SAM-future scores in predicting scores on the Cognitive Failures Questionnaire (CFQ)

	β	*SE*	*t*	*p*
Age	0.01	0.03	0.39	.70
SAM-future	-0.21	0.03	-6.78	< .001*
Age × SAM-future	0.02	0.03	0.72	.46

Note. SAM = Survey of Autobiographical Memory. *$p < .05$.

We conducted exploratory analyses to rule out alternative explanations for our main finding that individuals with lower trait episodic autobiographical memory (AM) did not report an age-related decrease in everyday functioning, relative to individuals with higher trait episodic AM. One such explanation is that low-episodic AM individuals cannot recall, and are thus unaware of, any deficits in daily function; as such, perhaps they objectively demonstrate just as much age-related decline as high-episodic AM individuals do, but do not report any complaints. To address this argument, we tested whether low-episodic AM individuals demonstrated any deficits in our objective measures of memory and cognitive performance.

First, we used a multilevel model to predict memory scores on the face-name recognition task as a function of age, recognition type (i.e., item versus associative), and SAM-episodic scores, as well as all possible interactions between these variables. The full results of the model are shown in Table S4.

Table S4

Parameters for multilevel linear model of age and SAM-episodic scores in predicting scores on the face-name memory task

	F	df	p	R^2
Age	3.64	1, 955	.06	.004
Recognition type	3.35	1, 955	.07	.003
SAM-episodic	0.54	1, 955	.46	.0006
Age × recognition type	5.50	1, 955	.02*	.006
Age × SAM-episodic	0.89	1, 955	.35	.0009
Recognition type × SAM-episodic	2.60	1, 955	.11	.003
Age × recognition type × SAM-episodic	2.70	1, 955	.10	.003

Note. SAM = Survey of Autobiographical Memory. *$p < .05$.

SAM-episodic scores were not directly related to memory scores nor did they interact with any of our other key predictors, indicating that individuals across the range of trait episodic AM abilities did not differ from each other in terms of performance on the face-name task. There was a statistically significant interaction between age and face-name recognition type, aligning with the results reported in the main text.

Next, we examined performance on each of the four CBS tasks: paired associates (Table S5), grammatical reasoning (Table S6), rotations (Table S7), and odd one out (Table S8). Again, SAM-episodic scores were not directly linked to performance on any of these tasks, nor did they interact with SAM-episodic scores to predict cognitive performance.

Table S5

Regression parameters for model of age and SAM-episodic scores in predicting scores on the Cambridge Brain Sciences (CBS) paired associates task

	β	*SE*	*t*	*p*
Age	-0.29	0.05	-6.23	< .001*
SAM-episodic	0.03	0.05	0.62	.53
Age × SAM-episodic	-0.04	0.05	-0.82	.41

Note. SAM = Survey of Autobiographical Memory. *$p < .05$.

Table S6

Regression parameters for model of age and SAM-episodic scores in predicting scores on the Cambridge Brain Sciences (CBS) grammatical reasoning task

	β	*SE*	*t*	*p*
Age	-0.36	0.04	-8.13	< .001*
SAM-episodic	-0.04	0.04	-0.90	.37
Age × SAM-episodic	-0.05	0.05	-1.17	.25

Note. SAM = Survey of Autobiographical Memory. *$p < .05$.

Table S7

Regression parameters for model of age and SAM-episodic scores in predicting scores on the Cambridge Brain Sciences (CBS) rotations task

	β	*SE*	*t*	*p*
Age	-0.13	0.05	-2.81	.005*
SAM-episodic	0.03	0.05	0.57	.57
Age × SAM-episodic	-0.01	0.05	-0.21	.83

Note. SAM = Survey of Autobiographical Memory. **p* < .05.

Table S8

Regression parameters for model of age and SAM-episodic scores in predicting scores on the Cambridge Brain Sciences (CBS) odd one out task

	β	*SE*	*t*	*p*
Age	-0.21	0.05	-4.47	< .001*
SAM-episodic	-0.04	0.05	-0.81	.42
Age × SAM-episodic	0.03	0.05	0.56	.57

Note. SAM = Survey of Autobiographical Memory. **p* < .05.

Finally, we explored whether there were gender differences in the interaction between age and SAM-episodic scores in predicting everyday cognitive function by including gender as a covariate in the linear regression. Omnibus results were obtained using Type III F-tests and are shown in Table S9. The relationship between age and daily function differed between males and females (Figure S1), but the interaction between episodic AM abilities and age in predicting everyday function did not differ by gender. While these exploratory results reveal interesting and potentially important gender differences, it is beyond the scope of the current article to interpret and discuss them in depth.

Table S9

Regression parameters for model of age, gender, and SAM-episodic scores in predicting scores on the Cognitive Failures Questionnaire (CFQ)

	df	*F*	*p*
Age	1	1.59	.21
SAM-episodic	1	33.92	< .001*
Gender	1	0.15	.70
Age × SAM-episodic	1	7.39	.001*
Age × gender	1	6.81	.009*
SAM-episodic × gender	1	0.10	.75
Age × SAM-episodic × gender	1	0.26	.61

Note. SAM = Survey of Autobiographical Memory. $^*p < .05$.

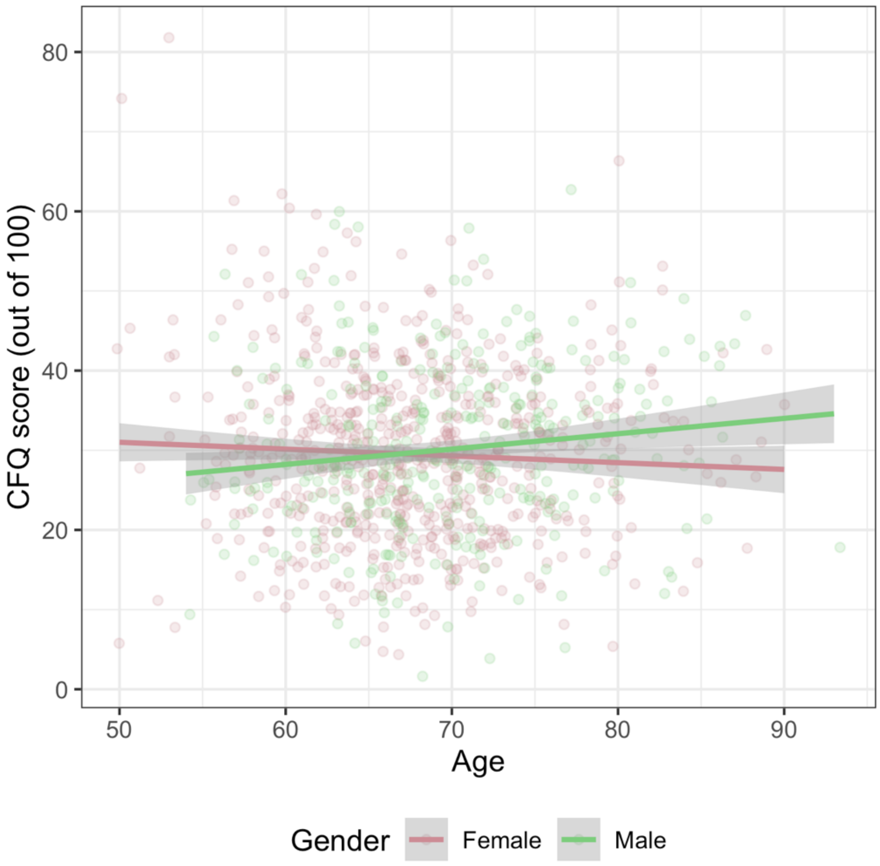

Figure S1. Scatterplot indicating a gender difference in the relationship between age and self-reported cognitive function in everyday life. Abbreviations: CFQ = Cognitive Failures Questionnaire. Higher CFQ scores indicate more complaints.

Overall, these results indicate that participants across the range of trait episodic AM performed similarly on our measures of memory and cognitive performance, and they suggest that low-episodic AM individuals did not appear to have objective cognitive impairments that would have led to a lack of awareness regarding general daily functioning.
